# Analysis of a radiographic parameter K-line tilt following adjacent two-level anterior cervical discectomy and fusion: a retrospective study

**DOI:** 10.1186/s13018-020-01639-0

**Published:** 2020-04-07

**Authors:** Zhibin Lan, Zhiqiang Wu, Weihong Xu, Yuming Huang

**Affiliations:** 1 Spinal Surgery Department, Quanzhou Orthopedic-Traumatological Hospital of Fujian Traditional Chinese Medicine University, Quanzhou, Fujian China; 2 Department of Spine Surgery, The First Affiliated Hospital of Fujian Medical University, Fuzhou, Fujian China; 3 The Orthopedics Department, Fuzhou Second Hospital affiliated to Xiamen University, Fuzhou, Fujian China

**Keywords:** K-line tilt, ACDF, Cervical sagittal alignment, SVA, NDI, T1 slope

## Abstract

**Background:**

T1s, C2-7 lordosis, and C2-7 sagittal vertical axis (SVA) are the three most important sagittal parameters in the cervical spine. This study was conducted to investigate the relationships between classical sagittal alignment parameters and a new parameter, K-line tilt, and to study the impact of K-line tilt.

**Material and methods:**

A total of 72 patients who underwent adjacent two-level anterior cervical discectomy and fusion (ACDF) were retrospectively analyzed. Radiographic measurements included T1 slope (T1s), C2-7 lordosis, segment angle (SA), C2-7 SVA, and K-line tilt. The Neck Disability Index (NDI) scores were used to evaluate the clinical prognosis. Pearson correlation coefficients were calculated between radiographic measures. Linear regression analysis was used to analyze the relationship between follow-up K-line tilt and NDI.

**Results:**

ΔNDI was positively correlated with ΔT1s (*r* = 0.620, *p* < 0.05), ΔC2-7 SVA (*r* = 0.645, *p* < 0.05), and ΔK-line tilt (*r* = 0.702, *p* < 0.01); ΔK-line tilt was positively correlated with ΔT1s (*r* = 0.650, *p* < 0.05), ΔSA (*r* = 0.269, *p* < 0.05), and ΔC2-7 SVA (*r* = 0.293, *p* < 0.05); ΔT1s was positively correlated with ΔC2-7 lordosis (*r* = 0.428, *p* < 0.05), ΔSA (*r* = 0.631, *p* < 0.01), and ΔC2-7 SVA (*r* = 0.235, *p* < 0.05); ΔC2-7 lordosis was positively correlated with ΔSA (*r* = 0.666, *p* < 0.05) and negatively correlated with ΔC2-7 SVA (*r* = − 0.467, *p* < 0.01). The preoperative and postoperative K-line tilt values were statistically significant (*p* < 0.01), increasing from (7.50 ± 6.48)° to (9.95 ± 5.09)°. Preoperative NDI was positively correlated with preoperative C2-7 SVA (*r* = 0.639, *p* = 0.011) and K-line tilt (*r* = 0.516, *p* = 0.026); follow-up NDI was positively correlated with follow-up T1s (*r* = 0.664, *p* = 0.038), C2-7 SVA (*r* = 0.756, *p* = 0.004), and K-line tilt (*r* = 0.832, *p* = 0.006). The linear regression model showed that when the follow-up K-lint tilt was > 23.75°, NDI scores were > 25 (*R*^2^ = 0.737, *p* = 0.000).

**Conclusion:**

This study showed that the K-line tilt was strongly correlated with the C2-C7 SVA, indicating that the K-line tilt can be used as another cervical parameter to evaluate cervical alignment in adjacent two-level ACDF. K-line tilt is an important parameter similar to the classical parameter C2–C7 SVA. In particular, a K-line tilt greater than 23.75 corresponded to a worse clinical prognosis, which was defined as an NDI score greater than 25.

## Background

Many studies have indicated that the sagittal balance of the cervical spine may play an important role in clinical prognosis [1–4]. Additionally, an imbalance in the sagittal plane of the cervical spine leads to an increase in Neck Disability Index (NDI) scores [5].

Kwon et al. [6] noted that a C2–C7 sagittal vertical axis (SVA) value of 40 mm was the cutoff score beyond which the NDI was vastly adversely affected. Weng et al. [3] conducted a study on patients with degenerative cervical disease and found that T1 slope (T1s), measured at the vertebral body at the intersection of the cervical spine and thoracic spine, had a significantly greater influence than the C2-7 SVA on the prognosis of patients. T1s may affect the curvature of the cervical spine to ensure that the center of gravity of the head is in a balanced position. In addition to the C2-7 SVA and T1s, other common traditional cervical sagittal plane parameters include C2-7 lordosis, segment angle (SA), neck tilt, and thoracic inlet angle.

The K-line was first described by Fujiyoshi et al. [7] as a line that connects the centers of the C2 and C7 spinal canals. This line is widely used in surgical approaches in patients with ossification of the posterior longitudinal ligament (OPLL). Fujiyoshi et al. divided cervical OPLL patients into two groups according to the position of the K-line, including a K-line (+) group, in which the OPLL did not exceed the K-line, and a K-line (−) group, in which the OPLL did exceed the K-line. The K-line (−) group did not exhibit satisfactory spinal cord repositioning and showed no obvious improvement in neurological function [7].

Recently, Kim et al. [8] were the first to report that K-line tilt is as important as the more traditional cervical sagittal parameter, SVA. K-line tilt is consistent with SVA, indicating a forward tilting state of the patient, in which the energy consumption of the rear muscle is increased, resulting in a poor clinical prognosis. The Pearson’s correlation coefficient between the K-line tilt and C2–C7 SVA was 0.813 (*p* = 0.000) [8]. However, no study has verified this finding, and there has been no clinical research regarding the correction of a patient’s K-line tilt. In this study, we examined the importance of K-line tilt in clinical data from patients who underwent adjacent two-level anterior cervical discectomy and fusion (ACDF) and evaluated the correlation between K-line tilt and NDI scores.

## Materials and methods

### Patient population

This work was approved by the institutional review board of our hospital. All methods were carried out in accordance with relevant guidelines and regulations. Informed consent was obtained from all patients (all above 18 years old). After obtaining approval from the institutional review board, the clinical and radiographic results of patients who underwent adjacent two-level ACDF of the lower cervical spine at the Department of Spine Surgery between January 2010 and December 2015 were retrospectively analyzed. All patients were diagnosed via a detailed inquiry of their medical history, a physical examination, and an imaging examination. The following inclusion criteria were applied: (1) preoperative (within 1 week before surgery), postoperative (within 3 days after surgery), and follow-up (at least 1 year after surgery) cervical X-ray films were available; (2) patients did not undergo other cervical spine surgery or cervical spine fixation; (3) signs and symptoms of spinal cord injury or nerve root damage; and (4) imaging suggested that cervical disc disease caused cervical spinal stenosis. Patients with trauma, tumor, spinal deformity, spinal infection, or severe osteoporosis were excluded. Patients in whom it was difficult to measure the sagittal alignment parameters (the T1 vertebral body could not be clearly seen on the X-ray, and the measurement of the vertebral body was blocked by the sternum or ribs on the sagittal plane) were also excluded.

### Radiologic parameters

**Fig. 1:**
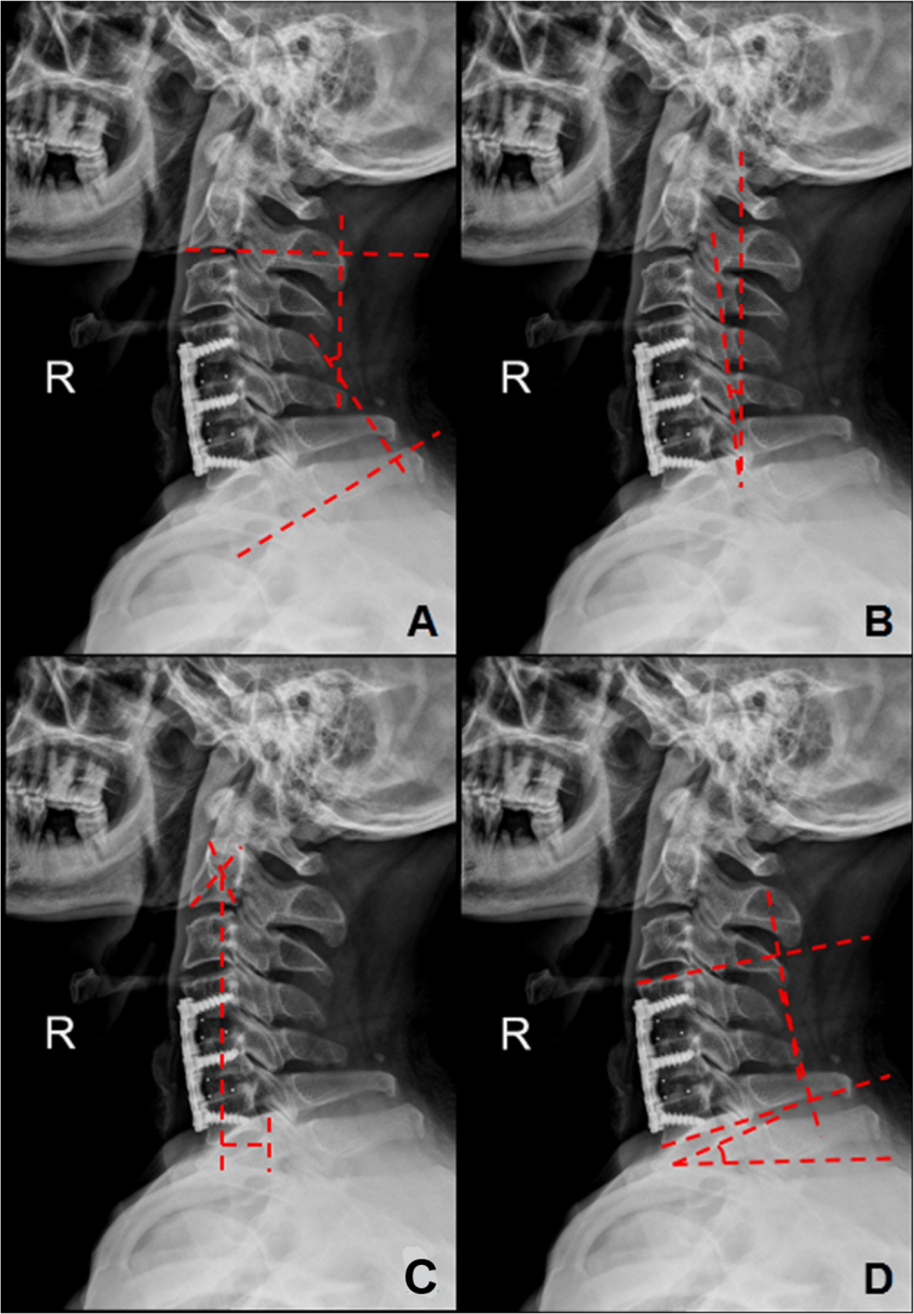
Measurement of parameters. **a** C2–7 lordosis. **b** K-line tilt. **c** C2–7 SVA, sagittal vertical axis. **d** SA, segment angle; T1s, T1 slope

A standard cervical X-ray series was obtained and uploaded to our Picture Archiving and Communication Systems (PACS) system. To obtain a lateral radiograph, the patient was told to stand upright as far as possible and look straight ahead. The following parameters were examined (Fig. 1): (1) T1s [9]: the angle between a horizontal line and the superior endplate of T1; (2) C2-7 lordosis [1]: the angle created by a line parallel to the inferior aspect of the C2 body and a line parallel to that of the C7 body was measured on neutral lateral radiographs; (3) SA: the angle created by two lines respectively parallel to the superior and inferior aspect of the surgical segmental body was measured on neutral lateral radiographs; (4) C2-7 SVA [1]: the distance between the C2 plumb line and the posterior superior endplate of C7, with positive sagittal alignment defined as an anterior deviation; (5) K-line tilt [8]: the angle between the K-line and a line perpendicular to the horizon. The difference between preoperative and postoperative values for each parameter was designated the Δ value. NDI scores were categorized as follows: 0–20, slight dysfunction; 21–40, moderate dysfunction; 41–60, severe dysfunction; 61–80, very severe dysfunction; and 81–100, full dysfunction or requiring a detailed examination in subjects with and without exaggerated symptoms. The clinical prognosis was assessed by health-related quality of life (HRQOL) surveys, and the NDI score was obtained for each patient at the time of their preoperative (within 1 week before surgery) and last follow-up office visits.

### Statistical analysis

SPSS 20.0 software (IBM Corp, New York, America) was used for all statistical analyses. Measurement data are shown as ‾x ± s. The Pearson correlation coefficient was calculated for between-time-point changes in X-ray measurements. Linear regression analysis was used to analyze the relationship between sagittal parameters in the cervical spine and NDI scores. A paired *t* test was used to evaluate the influence of the lateral position of the lower cervical spine on the sagittal position of the cervical spine. A value of *p* < 0.05 was defined as statistically significant.

## Results

### Demographic data

Overall, we reviewed clinical data from 75 patients, 1 of whom was excluded because of traumatic injury and 2 of whom were excluded because T1 could not be measured. A total of 72 patients (M/F = 46/26) were included, and their mean age was 52.6 ± 5.2 (range, 40–68) years. The following segments were involved: C3–5 (12 cases), C4–6 (39 cases), and C5–7 (21 cases). The average follow-up period for which postoperative radiographic measurements and HRQOL scores were obtained was 25.8 ± 7.6 months.

### Radiographic measurements and correlations

Table 1 summarizes the preoperative and postoperative and follow-up values obtained for radiographic measurements and comparisons among them.

**Table 1:** Effects on cervical sagittal alignment parameters following adjacent two-level ACDF

Item	Preoperative	Postoperative	Follow-up	*p* value	p* value
T1s (°)	27.78 ± 8.70	28.24 ± 6.67	27.61 ± 9.71	0.573	0.841
C2–7 lordosis (°)	17.80 ± 9.72	18.62 ± 6.84	17.42 ± 8.42	0.430	0.723
SA (°)	3.62 ± 9.14	9.68 ± 4.28	7.73 ± 4.90	0.000	0.000
C2–7 SVA (cm)	2.40 ± 1.86	2.65 ± 1.12	2.43 ± 1.64	0.236	0.918
K-line tilt (°)	7.50 ± 6.48	9.95 ± 5.09	9.49 ± 8.95	0.000	0.001

*p value* comparison between preoperative and postoperative, p* value comparison between preoperative and follow-up, *T1s* T1 slope, *SA* segmental angle, *C2-7SVA* sagittal vertical axis

Pearson’s correlation coefficient was calculated for changes in radiographic measures: ΔNDI was positively correlated with ΔT1s (*r* = 0.620, *p* < 0.05), ΔC2-7 SVA (*r* = 0.645, *p* < 0.05) and ΔK-line tilt (*r* = 0.702, *p* < 0.01); ΔK-line tilt was positively correlated with ΔT1s (*r* = 0.650, *p* < 0.05), ΔSA (*r* = 0.269, *p* < 0.05), and ΔC2-7 SVA (*r* = 0.293, *p* < 0.05); ΔT1s was positively correlated with ΔC2-7 lordosis (*r* = 0.428, *p* < 0.05), ΔSA (*r* = 0.631, *p* < 0.01), and ΔC2-7 SVA (*r* = 0.235, *p* < 0.05); and ΔC2-7 lordosis was positively correlated with ΔSA (*r* = 0.666, *p* < 0.05) and negatively correlated with ΔC2-7 SVA (*r* = − 0.467, *p* < 0.01) (Table 2).

**Table 2:** Correlations between the changes of sagittal alignment parameters following adjacent two-level ACDF

		ΔT1s	ΔC2–7 lordosis	ΔSA	ΔC2–7 SVA	ΔK-line tilt	ΔNDI
ΔT1s	*r*	1	.428^*^	.631^**^	.235^*^	.650^*^	.620^*^
	*p*		.037	.005	.047	.042	.086
ΔC2–7 lordosis	*r*	.428^*^	1	.666^*^	− .467^**^	-.023	.102
	*p*	.037		.022	.009	.847	.382
ΔSA	*r*	.631^**^	.666^*^	1	− .121	.269^*^	− .080
	*p*	.005	.022		.312	.026	.592
ΔC2-7SVA	*r*	.235^*^	− .467^**^	− .121	1	.293^*^	.645^*^
	*p*	.047	.009	.312		.013	.048
ΔK–line tilt	*r*	.650^*^	− .023	.269^*^	.293^*^	1	.702^**^
	*p*	.042	.847	.026	.013		.028
ΔNDI	*r*	.620^*^	.102	− .080	.645^*^	.702^**^	1
	*p*	.086	.382	.592	.048	.028	

*T1s* T1 slope, *SVA* sagittal vertical axis, *SA* segmental angle, *NDI* neck disability index

^*^Correlation is significant at the *p* < 0.05 level (2-tailed)

^**^Correlation is significant at the *p* < 0.01 level (2-tailed)

### Correlations between NDI and parameters

**Fig. 2:**
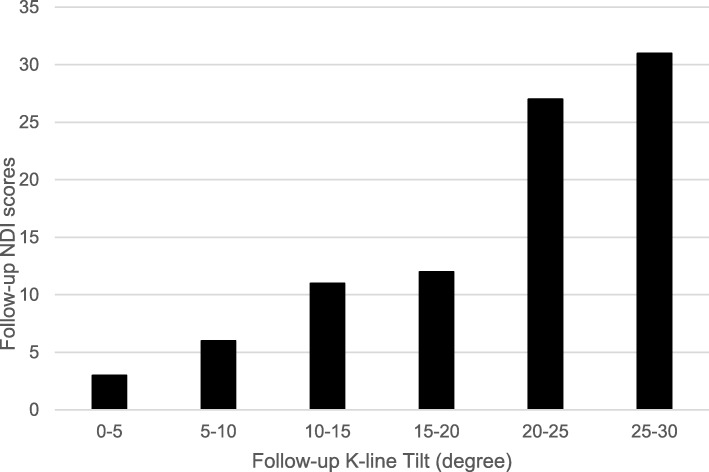
Bar graph of mean data showing a positive correlation between follow-up K-line tilt and NDI scores. NDI, neck disability index

Comparisons between radiographic measurements and NDI scores demonstrated a significant positive correlation between K-line tilt values and NDI scores (Fig. 2).

**Fig. 3:**
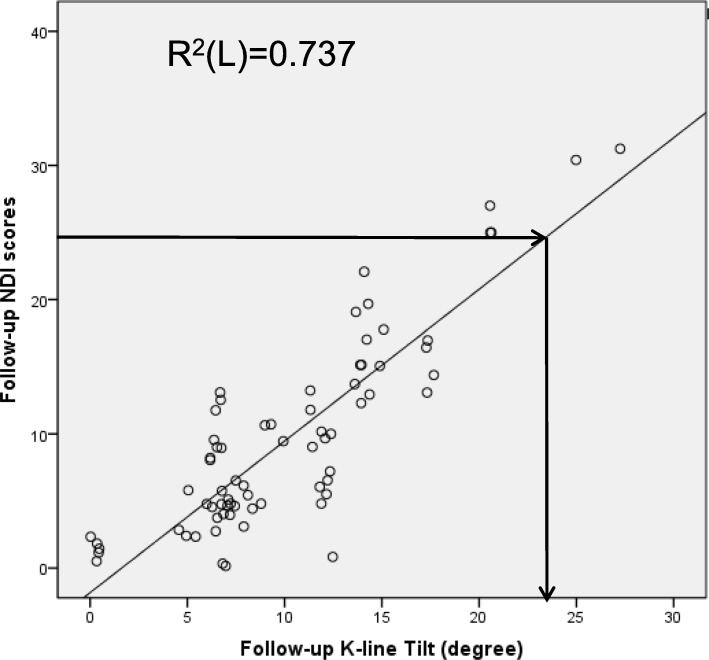
The linear regression model of follow-up K-line tilt and NDI scores showed that when K-line tilt was > 23.75°. NDI scores were > 25 (*R*^2^ = 0.737, *p* = 0.000). NDI, neck disability index

Preoperative NDI scores were positively correlated with preoperative C2-7SVA (*r* = 0.639, *p* = 0.011) and K-line tilt (*r* = 0.516, *p* = 0.026), and follow-up NDI scores were positively correlated with follow-up T1s (*r* = 0.664, *p* = 0.038), C2-7 SVA (*r* = 0.756, *p* = 0.004), and K-line tilt (*r* = 0.832, *p* = 0.006). The linear regression model showed that when the follow-up K-line tilt was > 23.75°, NDI scores were > 25 (*R*^2^ = 0.737, *p* = 0.000) (Table 3) (Fig. 3).

**Table 3:** Correlations between sagittal parameters and NDI scores

	Sagittal parameters	Correlation (Spearman *r*)	*p* value
Preoperative NDI scores	Preoperative T1s	0.652	0.072
	Preoperative C2–7 lordosis	0.090	0.451
	Preoperative SA	-0.078	0.517
	Preoperative C2–7 SVA	0.639^*^	0.011
	Preoperative K-line tilt	0.516^*^	0.026
Follow-up NDI scores	Follow-up T1s	0.664^*^	0.038
	Follow-up C2–7 lordosis	0.117	0.326
	Follow-up SA	-0.065	0.585
	Follow-up C2–7 SVA	0.756^**^	0.004
	Follow-up K-line tilt	0.832^**^	0.006

*NDI* neck disability index, *T1s* T1 slope, *SA* segment angle, *SVA* sagittal vertical axis

^*^Correlation is significant at the *p* < 0.05 level (2-tailed)

^**^Correlation is significant at the *p* < 0.01 level (2-tailed)

## Discussion

In recent years, concern has increased regarding the importance of cervical sagittal parameters in clinical prognoses [1, 4, 5]. Therefore, accurately and rapidly evaluating cervical sagittal balance requires good cervical parameters.

We found that T1s, C2–7 lordosis and C2–7 SVA were the three most important sagittal parameters in the cervical spine. A study by Kim et al. [4] showed that patients with a large T1s required increased cervical lordosis and increased energy consumption in the upper cervical spine to maintain their head weight. A study by Oe et al. [10] showed that when T1s > 40°, the probability of failure was higher for deformity corrections performed in the cervical vertebra. Many studies have also noted that maintaining surgical intervention for C2–7 lordosis has a positive effect on a patient’s prognosis, perhaps because less energy is consumed by the neck muscles and ligaments [11–13]. Tang et al. [5] also suggested that when C2–7 SVA > 40 mm, NDI scores will be worse.

In this study, from Table 1, we could conclude that the surgery mainly changed the SA (*p* = 0.000) and K-line tilt (*p* = 0.000), but by analyzing the correlations among Δ values (Table 2), we concluded that surgery indirectly changed other sagittal parameters by directly changing the SA and K-line tilt. These data clearly indicated that ACDF surgery maintains global lordosis mainly by increasing regional lordosis.

Among the indicators measured, the correlation between C2–7 lordosis and NDI scores was not obviously significant. The T1 vertebral body is not a direct surgical segment because it cannot be directly changed by surgery but must instead be indirectly changed. These changes were relatively small. The good cervical sagittal observation index can evaluate the curvature of the entire cervical vertebrae. As the most active cervical vertebra, it is impossible to display the complete cervical vertebrae by simply evaluating T1s, but the K-line tilt (a line connecting the centers of the C2 and C7 spinal canals) can better evaluate sagittal balance. Furthermore, the T1s is not easy to measure because the thoracic spine is difficult to correctly identify on a simple lateral X-ray film due to anatomical interference from the shoulder contour density, especially in obese people with thick thoraces [14]. Although T1s minus C2–7 lordosis (T1s-CL) is also a good cervical parameter, it is more difficult to measure and use. The K-line can be measured by identifying and connecting the two midpoints of the C2 and C7 spinal canals. Therefore, K-line tilt detection is simple and convenient and can be performed quickly and intuitively.

Of the indicators evaluated in our study, we found that both K-line tilt and C2–7 SVA were substantially correlated with NDI scores (*r* = 0.832 vs 0.756), and K-line tilt was positively correlated with C2–7 SVA (*r* = 0.707, *p* = 0.008) and T1s (*r* = 0.501, *p* = 0.036). During the operation, the angle is more intuitive than the length because there is no need to consider the effect of scale. Therefore, we believe that K-line tilt may provide a more advantageous assessment method. Linear regression analysis was used to analyze the relationship between K-line tilt and NDI scores, and the results indicated that when the follow-up K-line tilt was > 23.75°, the NDI scores were > 25 (*R*^2^ = 0.737, *p* = 0.000). In this regard, we believe that if the patient’s K-line tilt is larger before surgery, it may be considered to increase the size of the cage (5, 6, and 7 mm are commonly used) during surgery and to correct the regional lordosis, which will yield a better prognosis for the patient.

This study only included a small number of sample statistics. Moreover, this study was retrospective, and therefore, some unintended biases could exist, such as selection bias and information bias. NDI scores were used to evaluate the clinical prognosis and as a quality of life index, and the JOA scores, SF-36, and VAS scores were not measured. We chose adjacent two-level ACDF because the single-segment surgery (single-segment ACCF or disc replacement, etc.) resulted in little change in sagittal parameters before and after surgery. There were more patients with two-level ACDF in our hospital. In principle, if there are sufficient segments to perform multi-segment ACDF, this approach is best. In this study, we only had one experienced doctor to measure the data, which inevitably leads to errors. We hope that a follow-up study will enhance and verify the value of this article by increasing the sample size and improving scoring standards.

## Conclusion

This study showed that the K-line tilt was strongly correlated with the C2–C7 SVA, indicating that the K-line tilt could be used as another cervical parameter to evaluate cervical alignment in adjacent two-level ACDF. The K-line tilt was also an important parameter, similar to the classical parameter C2–C7 SVA. In particular, a K-line tilt greater than 23.75 corresponded to a worse clinical prognosis, which was defined as an NDI score greater than 25.

## Data Availability

To preserve the privacy of the patients, their clinical data will not be shared; data can be available from authors upon request.

## Abbreviations

ACDF: Anterior cervical discectomy and fusion
HRQOL: Health-related quality of life
NDI: Neck disability index
SA: Segment angle
SVA: Sagittal vertical axis
T1s: T1 slope
